# Meteorological Table

**Published:** 1812-04

**Authors:** 


### METEOROLOGICAL TABLE.

### *From February 26, to March 26, 1812.*

| | D. | Therm. | | | Barom. | | Hygrom. *Dry.——Damp.* | | | | Winds. | Atmos. Variation. |
|---|---|---|---|---|---|---|---|---|---|---|---|---|
| ○ | 27 | 37 | 42 | 40 | $29^{7}$ | 6 | | 25 | — | — | S.. | F.. R . F .... |
| | 28 | 38 | 44 | 40 | $29^{7}$ | 6 | | 23 | 16 | 32 | W. | F ... — .... — .... |
| | 29 | 37 | 44 | 42 | $29^{5}$ | 4 | | 25 | 20 | 22 | S.. | C .. F ... R . F.... |
| | 1 | 42 | 45 | 40 | $29^{4}$ | 6 | | 25 | 16 | 22 | NE..E. | C ... F .... — ... |
| | 2 | 37 | 42 | 37 | $29^{8}$ | 9 | | 22 | 10 | 16 | NE.. | F... — ... H .. F .... |
| | 3 | 35 | 40 | — | $29^{9}$ | 7 | | 15 | 17 | 22 | S.SW.. | C...F..R..—.—... in N |
| | 4 | 42 | 50 | 46 | $29^{7}$ | — | | 27 | 28 | 30 | SW.. | C .... R .. — . F .... |
| | 5 | 40 | 46 | 41 | $29^{8}$ | 30 | | 26 | 4 | 15 | NW.. | F... — .... — ... |
| ◑ | 6 | 41 | 50 | 45 | $29^{6}$ | 8 | | 27 | 15 | 20 | NW.. | R .. F ... — ... C ... |
| | 7 | 48 | 53 | 44 | $29^{8}$ | 6 | | 30 | 21 | 24 | W.. | F .. C .... R .. C .... |
| | 8 | 41 | 46 | 39 | $29^{8}$ | $30^{1}$ | | 22 | 15 | 20 | NW..N.. | F.. R . — .. F ... |
| | 9 | 36 | 42 | 38 | $30^{2}$ | 3 | | 20 | 8 | 16 | N.. | F... S . F .... |
| | 10 | 38 | 45 | 38 | $30^{3}$ | 2 | | 16 | 2 | 12 | NE.. | C ... F .... — .... |
| | 11 | 37 | 46 | 40 | $30^{2}$ | | | 13 | 5 | 11 | NE.. | C ... — ... — .... |
| | 12 | 38 | 45 | 39 | $30^{2}$ | $29^{9}$ | | 14 | 6 | 15 | N.NW.W. | C... F ... R .. |
| ● | 13 | 37 | 44 | 39 | $29^{8}$ | 9 | | 11 | 5 | 13 | N.. | F.... — ... R . S . R. |
| | 14 | 39 | 48 | 38 | 30 | $29^{9}$ | | 13 | 12 | 13 | NE.. | F .... R .. F .... |
| | 15 | 35 | 43 | 35 | $29^{8}$ | 7 | —1— | 12 | — | 10 | NE. | F .... — .... — .... |
| | 16 | 35 | 39 | 34 | $29^{7}$ | 6 | | 10 | 3 | 9 | NE... | C ... — ... S . C .... |
| | 17 | 35 | 38 | 34 | $29^{7}$ | — | | 5 | — | 8 | NE.. | S . C .... S . C .... |
| | 18 | 33 | 38 | 35 | $29^{5}$ | 3 | —1— | 7 | — | 7 | NE. | F... C ... — .... |
| ◐ | 19 | 34 | 38 | 36 | $29^{3}$ | | | 7 | 1 | 7 | E.. | F.. C ... — ... |
| | 20 | 36 | 42 | 44 | $29^{2}$ | 29 | | 11 | 13 | — | SE.. | S.. R .. C .... |
| | 21 | 43 | 52 | 45 | $29^{1}$ | 3 | —2— | 16 | — | 8 | SW... | F... — .... R. — .. in N |
| | 22 | 44 | 50 | 44 | $29^{4}$ | 5 | | 10 | 7 | 10 | SE.. | C ... — .. F . C ... |
| | 23 | 43 | 44 | 42 | $29^{7}$ | 3 | | 15 | 13 | 10 | E.. | C .... —...R...—... in N |
| | 24 | 43 | 42 | 38 | $29^{1}$ | 4 | | 19 | 12 | 16 | E.. | C.... R.. — .. S.. in N |
| | 25 | 36 | 39 | 32 | $29^{5}$ | $30^{1}$ | | 15 | 2 | 10 | NE.. | F.. — .. S . F .... |
| | 26 | 31 | 43 | 37 | $30^{2}$ | 3 | | 13 | 3 | 9 | SE. | F.... — .... — .... |

Quantity of rain from February the 26th to March the 26th, 1 inch $\frac{52}{100}$.

The range of the thermometer has been very great, from 53 on the 7th, to 31 on the 26th. The degree of humidity, as indicated by the hygrometer, has been considerably less than in February. The prevailing winds have been from the N. and N.E. Nineteen days in this interval, it has blown from these quarters.

On the first, though fair, the sensation of cold was peculiarly unpleasant.

On the 2d, also fair, but abstractive of caloric in a much greater proportion than the state of the thermometer indicated.

On the 4th, raining lightly from noon yesterday, through the whole of the night. This approach to rain was not indicated by increase of humidity in the atmosphere; on the contrary, the hygrometer pointed yesterday morning to a higher degree of dryness than had occurred within the last month. The humidity increasing with the fall of rain; but this is not, as might be expected, an invariable occurrence. Temperature remarkably soft. The terms hard and soft, as applied to states of the atmospheric fluid, are not to be considered as loose and popular only; they are faithfully descriptive of qualities in the air, independent of the velocity of winds, they actually impinge on surfaces with a peculiarity, at times, that gives the idea of the current being hard; while, at other times, a sense of softness is equally palpable. It will generally happen, however, ever, though not invariably, that the sensation excited of softness, will accompany high temperature and humidity, and hardness that of cold and dryness.—5th, Evaporation rapid.—7th, Warm, with clouds and misty rain.—8th, Snowing.—9th, Showers of snow, cold and stormy. Barometer high, and air dry.—16th, Wind for several days from N. and N. E. and accompanied with unpleasantly cold sensation. During the prevalence of these winds, when the air is dry, atmospherical electricity is at a low standard; its existence, sometimes, not detectable by the most sensible electrometer. Does the peculiar quality of the east wind depend on this, and does its unhealthiness arise from the same cause? Cold and dryness, accompanied with rapid evaporation, probably is, of all other states of the atmosphere, most dangerous to the bronchial membrane, and even to the parenchemia of the lungs; but what share the low quantum of electric fluid has in the production of pulmonic complaints, is a question that remains to be solved; and the inquiry is not uninteresting.—20th, Snow in the night, and continuing this morning, covering the streets and houses, but rapidly dissolving and followed by rain.—25th, Thermometer very low, 31, at eight in the morning.

Pulmonic diseases have been frequent in this interval, and a catarrhal affection has existed very extensibly among horses throughout the metropolis.

*Princes'-street, Cavendish-square.*

